# Quantifying the energy stores of capital breeding humpback whales and income breeding sperm whales using historical whaling records

**DOI:** 10.1098/rsos.160290

**Published:** 2017-03-15

**Authors:** Lyn G. Irvine, Michele Thums, Christine E. Hanson, Clive R. McMahon, Mark A. Hindell

**Affiliations:** 1 Institute for Marine and Antarctic Studies, University of Tasmania, Hobart, Tasmania, Australia; 2 Australian Institute of Marine Science and the Indian Ocean Marine Research Centre, University of Western Australia (M096), Western Australia, Australia; 3 BMT Oceanica, Wembley, Western Australia, Australia; 4 Sydney Institute of Marine Science, Mosman, New South Wales, Australia

**Keywords:** bioenergetics, body condition, body lipid, oil yield, life history, cetacean

## Abstract

Cetacean energy stores are known to vary according to life history, reproductive status and time of year; however, the opportunity to quantify these relationships is rare. Using a unique set of historical whaling records from Western Australia (1952–1963), we investigated energy stores of large cetaceans with differing life histories, and quantified the relationship between total body lipid and length for humpback whales (*Megaptera novaeangliae)* (*n* = 905) and sperm whales *(Physeter macrocephalus)* (*n* = 1961). We found that total body lipid increased with body length in both humpback and sperm whales, consistent with size-related energy stores. Male humpback whales stored 2.49 kl (15.6 barrels) (31.9–74.9%) more lipid than male sperm whales of equivalent length, to fuel their annual migration. Relative lipid stores of sperm whales (males) were constant throughout the year, while those of humpback whales varied with reproductive class and sampling date. Pregnant female humpback whales had higher relative energy stores than non-pregnant females and males (26.2% and 37.4%, respectively), to fuel the energy demands of gestation and lactation. Those that reached the sampling site later (*en route* to their breeding grounds) carried higher lipid stores than those that arrived earlier, possibly reflecting individual variation in residency times in the Antarctic feeding grounds. Importantly, longer pregnant females had relatively larger energy stores than the shorter pregnant females, indicating that the smaller individuals may experience higher levels of energetic stress during the migration fast. The relationships we developed between body lipid and length can be used to inform bioenergetics and ecosystem models when such detailed information is not available.

## 1. Introduction

To optimize their fitness, organisms must acquire and allocate resources over their lifetime in a way that maximizes individual survival and reproduction [1]. A range of life-history strategies have evolved to achieve this, with ‘capital breeding’ and ‘income breeding’ representing the extremes of a continuum in how individuals store and allocate maternal resources [1,2]. Capital breeders use stored energy for reproduction and survival, while income breeders use energy that is acquired on a continual basis, including during the reproductive period [3,4]. Energy storage enables the temporal and spatial separation of different activities such as feeding and breeding [2] and hence utilization of environments where resources such as food may be temporarily abundant, but where physical conditions may be unsuitable for successful reproduction [5,6]. The capital breeding strategy generally involves a life cycle that consists of periods of intensive feeding and fasting that are synchronized with the annual seasonal cycle [7–10]. For reproductive females, this strategy requires short periods of maternal care with high rates of energy flow to the young [11–13]. The income breeding strategy of continuous energy acquisition throughout the year enables longer periods of maternal care [12,13], but requires stable or predictable environments.

Among mammals, the capital breeding strategy is restricted to large animals such as bears, true seals and baleen whales, due to the extreme energy demands of lactation during fasting [12,14] and relatively low mass-specific energy requirements of large body size [15]. Cetaceans are theoretically ideal candidates for investigating energy storage strategies for different life histories, as their considerable body size enables large energy stores relative to reproductive demands [14]. However, in practice, this large body size combined with their marine existence prevents capture for measurement, making quantification of cetacean energy stores extremely difficult.

Among the cetaceans, baleen whales are typically capital breeders while toothed whales are generally income breeders [13]. Many baleen whales, including humpbacks, move between highly productive polar feeding grounds during summer and relatively unproductive subtropical breeding grounds in winter [16]. This strategy requires the accumulation and storage of sufficient energy reserves to meet the cost of growth, maintenance, locomotion and reproduction in the breeding grounds [2]. As reproductive costs are highest for breeding females, due to energy-expensive lactation [14], they require larger energy stores than males and non-pregnant females [16–18]. By contrast, toothed whales typically meet their energy demands throughout the year by continual foraging [13,19] and thus have no need to store large energy reserves. Consequently, the energy stores of capital and income breeders should differ: capital breeders (particularly pregnant females) should have high energy stores on departure from feeding grounds, followed by a continual decline in energy stores throughout the migration and the reproductive cycle (e.g. [20]), until return to their feeding grounds; income breeders should have relatively constant energy stores throughout the year, given adequate food resources.

In cetaceans, energy is stored as lipid in various depots throughout the body, initially in the blubber, and then in the bone, muscle and viscera [16,21]. Cetacean energetics studies have typically focused on the blubber layer as it is an important and easily measured lipid store (e.g. [22,23]). However, lipid storage in body tissues other than the blubber can be substantial [15,24,25] and should therefore also be considered. For example, muscle is a major lipid depot in both blue and fin whales [16], and bone in fin whales can store nearly as much lipid as the blubber [26]. Furthermore, analyses of the blubber layer can also be complicated by differences in thickness and lipid content at different sites along the body [25,27–29], with no apparent correlation between the two [30]. To obtain a complete picture of energy storage, lipid stores in all body tissues must ideally be accounted for.

We recently located a historical whaling dataset from the Cheynes Beach Whaling Station, on the south coast of Western Australia. This whaling station, located about halfway along the migratory corridor of the Breeding Stock D (BSD) humpback whales [31], processed humpback whales that were hunted between 1952 and 1963 during their northward migration, and sperm whales hunted throughout the year between 1955 and 1976. Oil was extracted from the entire carcass (Bruce Teede, Engineer (retired) Carnarvon Whaling Station, Babbage Island, Carnarvon, Western Australia, August 2016, personal communication), and detailed records of oil yield, length and sex were recorded for the majority (94.7%) of individuals processed between 1952 and 1963. After this time, the station expanded and oil yields were reported as weekly tallies. The individual catch records from 1952–1963 provide a unique dataset of individual whale oil yield that can be used to quantify total body lipid stores of two large cetaceans. The records from 1953 and 1954 have been used previously to quantify the relationship between humpback whale oil yield and body length [32].

In this study, we extend these analyses [32] by using the individual catch records from Cheynes Beach Whaling Station to investigate how energy stores vary among cetaceans with different life histories. We compare and quantify total body lipid of humpback whales and sperm whales to test the hypothesis that energy stores of humpback whales are higher than those of sperm whales due to their different life-history strategies. We then investigate the variation in body lipid of each species separately, according to body length, reproductive class and time of year, and produce equations to quantify these relationships. We predict that the energy stores of the income breeding sperm whales will remain constant throughout the year, while that of the capital breeding humpback whales will vary with the energy demands of each reproductive class. Chittleborough [32] illustrated that pregnant female humpbacks had the largest energy stores due to the energy demands of pregnancy and lactation [16]. In addition to this, we predict that: (i) mature, non-pregnant females will have variable energy stores due to their varying reproductive states, i.e. some are recovering from lactation, while others are preparing for pregnancy; (ii) mature males will have higher energy stores than mature non-pregnant females, due to their need to compete for, and access, breeding females [33,34]; and (iii) immature whales will have higher energy stores than mature males and mature non-pregnant females due to the high energy demands of growth [35]. Furthermore, as humpback whales spend the summer accumulating and storing energy at a rate of 200 l per week [36] and all migrate at the same speed [37], we predict that individuals sampled at Cheynes Beach Whaling Station later in the season will have higher energy stores than those sampled earlier.

## 2. Material and methods

### 2.1. Data

**Figure 1.**
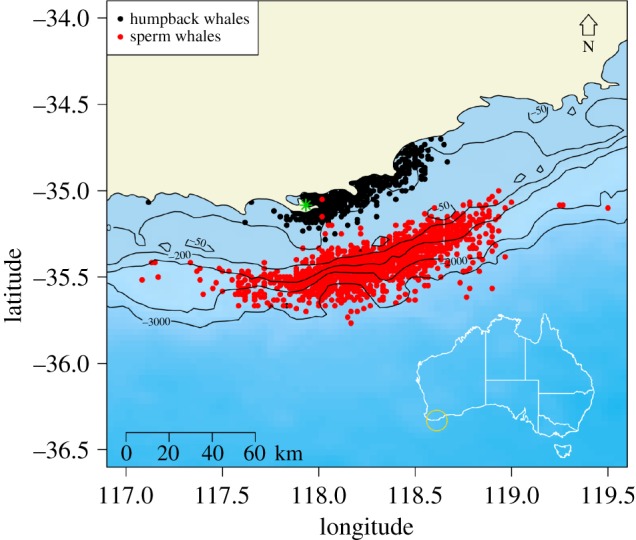
Location of Cheynes Beach Whaling Station (green asterisk), on the southwest coast of Western Australia, showing catch positions of humpback whales and sperm whales processed there between 1952 and 1963.

Data were sourced from catch records that detailed 3000 individual whales processed at Cheynes Beach Whaling Station (35°05′ S, 117°56′ E): 961 humpback whales caught between 1952 and 1963; 2039 sperm whales caught between 1955 and 1963 (electronic supplementary material, table S1 in appendix S1). Humpback whales were captured over the continental shelf, in waters generally less than 50 m deep (median = 13 m), while sperm whales were captured over the continental slope in waters generally 200–3000 m deep (median = 1067 m) (figure 1).

**Figure 2.**
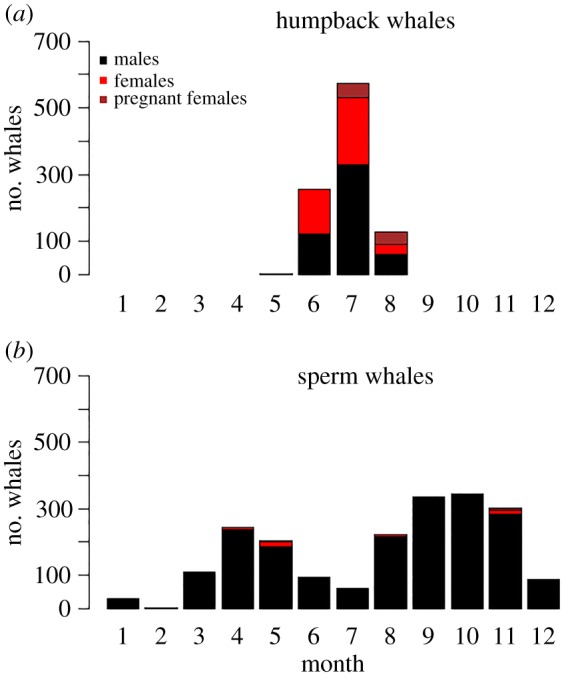
Number of (*a*) humpback whales and (*b*) sperm whales of each reproductive class (males, females and pregnant females) processed at Cheynes Beach Whaling Station each month during the years 1952–1963.

Humpback whales were caught between May and August, with highest catch numbers in July (figure 2). All humpbacks were caught on their northward migration between the Antarctic feeding grounds and lower latitude breeding grounds, as this population does not pass Cheynes Beach Whaling Station on their return journey to Antarctic waters [32]. Sperm whales were caught in all months of the year, with a small peak occurring between April and May and a larger peak between September and November (figure 2). Sperm whale catches over summer (December–February) and winter (June–July) were low (figure 2)—the winter catch being influenced by the local availability of humpback whales at this time of the year [38,39].

The catch records included individual details of catch date, location, whale length (recorded in feet), sex, oil yield (recorded in barrels) and length and sex of any fetus. We converted length into metres (1 ft = 0.3048 m) and oil yield into kilolitres (1 barrel = 0.16 kl [24]). All data were checked for potential ‘stretching’, whereby whalers reported an undersized whale as longer than the actual size to avoid an infraction of the minimum size regulation (35 feet or 10.7 m) [32,40]. We did this by constructing histograms of length and assessing the distribution to see if there was a notable peak around this minimum. Stretching was only identified for the female sperm whale data, and this was subsequently excluded from the analysis (accounting for 4% of total sperm whale data) (electronic supplementary material, figure S1 in appendix S2).

The measurement of total body lipid requires complete oil extraction from every part of the whale body. Oil extraction at Cheynes Beach Whaling Station was a two-step process carried out in large digesters. Initially, the carcass was flensed for oil extraction from the blubber; then the remainder of the carcass was sawn into pieces and rendered for oil extraction from the muscle, bone and viscera (Bruce Teede, Engineer (retired) Carnarvon Whaling Station, Babbage Island, Carnarvon, Western Australia, August 2016, personal communication). Once the oil was recovered, meat meal and whale solubles were produced from the remaining residue (raw products such as whale meat were not produced at the Cheynes Beach Whaling Station [41]). Owing to this extraction process, the oil yield records for sperm whales included sperm oil from the body as well as spermaceti oil from the spermaceti organ. The implications of this are discussed in a later section.

### 2.2. Statistical analyses

To examine the relationship in total body lipid (kl) and body length (m) between and within the humpback and sperm whales, we constructed a suite of linear mixed-effects models, including all combinations of the individual predictor variables (described below) and their interactions. Year was included as a random effect to account for potential inter-annual variation in oil yield. All models were fitted in R (R v. 3.0.2) [42] using the packages *nlme* [43] and *MuMIn* [44]. We compared and ranked each model in the suite with Akaike's information criterion corrected for small samples (AICc) and by their relative goodness-of-fit, the AICc weight (*w*AICc) [45,46]. The AIC weight varies from 0 (no support) to 1 (complete support) relative to all models in the set [46]. We also calculated the weights of the Bayesian information criterion (*w*BIC), and where the ranking did not agree with *w*AICc, we used *w*BIC for model selection (as AIC frequently prefers a more complex model) [47]. Where models were within 2 AIC or BIC points of one another, they were considered equal and, under the principle of parsimony, the simplest model (one with the least terms) was selected. The proportion of variance in the response variable, explained for fixed factors ($R_{\mathrm{GLMM}(m)}^{2}$) and fixed and random factors combined ($R_{\mathrm{GLMM}(\text{c})}^{2}$) [48], was used to quantify goodness-of-fit to the data [46].

#### 2.2.1. Inter-species differences

We tested for differences in lipid stores between species by modelling total body lipid as a function of species and length. We had to restrict this analysis to males due to the unreliable length measurements of the female sperm whales. We also restricted the analysis to the length range common to both species (10.7 m (35 ft)–14.2 m (46.5 ft)) and the years where both sperm and humpback whales were processed (1956–1962).

#### 2.2.2. Intra-species differences

##### 2.2.2.1. Humpback whales

To test for differences in lipid stores between reproductive classes, we modelled the relationship between total body lipid, length and reproductive class (males, pregnant and non-pregnant females), using linear mixed-effects models. Male humpback whales less than 11.2 m (36.75 ft) and females less than 11.7 m (38.5 ft) were classified as immature whales after Chittleborough [32], and colour coded in the plots as a separate reproductive class. They could not be analysed separately as the range in their body length did not cover the range in body length for the other classes. Given that the data were heterogeneous, with residuals increasing with body length, variance was weighted according to a power relationship of length [49].

Humpback whale analyses were restricted to the months June–August and the years 1953–1962. Data from 1952 was excluded as factory efficiency in the first year of production was typically lower than in subsequent years [32]; data from years 1954, 1963 and May were excluded due to low sample size (electronic supplementary material, table S1 in appendix S1). In 1955 body lengths were reported in feet only, rather than feet and inches as in all other years. To account for this, the dataset was analysed with and without the 1955 data. The results were the same with both analyses and thus the 1955 data were included in the final analysis.

To test our hypotheses regarding variation in energy stores through time, we modelled total body lipid as a function of length and month of catch. We analysed each reproductive class separately due to the temporally staggered migration of this population [32]. In addition, as the number of pregnant females and immature whales arriving in June and August, respectively, were low, (electronic supplementary material, table S1 in appendix S1), we restricted the analysis of the pregnant females to the months of July and August and that of immature whales to June and July. The lipid stores of the males and non-pregnant females were modelled over the full data range.

##### 2.2.2.2. Sperm whales

We examined the relationship between total body lipid, length and season for male sperm whales only as female sperm whales had unreliable length measurements (as mentioned above). We used season (summer = December–February, autumn = March–May, winter = June–August, spring = September–November) as a fixed effect in the models, rather than month (as in the humpback whale analyses) because sample sizes were not sufficient in all months (electronic supplementary material, table S1 in appendix S1). We restricted the analysis to 1956–1963 as the sample size in 1955 was too low (*n* = 4; electronic supplementary material, table S1 in appendix S1).

## 3. Results

The restricted dataset that we used for body lipid analyses contained detailed information for 905 humpback whales and 1961 sperm whales (table 1). Of the humpback whales, 39.6% were adult males (*n* = 358), 24.1% were non-pregnant females (*n* = 218), 8.1% were pregnant females (*n* = 73) and 28.3% were immature whales (*n* = 256). All of the sperm whales were adult males (*n* = 1961) (table 1).

**Table 1.** Mean and standard deviation length, total body lipid and energy stores of whales processed at Cheynes Beach Whaling Station for 1953–1963 (the following data were excluded from the analysis: humpback whale data from 1952, 1954, 1963 and May; sperm whale data from 1955).

	humpback whales				sperm whales			
reproductive group	*n*	length (m)	total body lipid (kl)	energy storage (kl m^−1^)	*n*	length (m)	total body lipid (kl)	energy storage (kl m^−1^)
males	358	12.08 ± 0.62	7.58 ± 1.26	0.63 ± 0.08	1961	13.27 ± 1.22	6.63 ± 1.63	0.49 ± 0.08
non- pregnant females	218	12.76 ± 0.75	8.71 ± 1.64	0.68 ± 0.11	n.a.	n.a.	n.a.	n.a.
pregnant females	73	13.07 ± 0.76	11.30 ± 2.63	0.86 ± 0.16	n.a.	n.a.	n.a.	n.a.
immature	256	10.94 ± 0.43	6.38 ± 0.96	0.58 ± 0.08	—	—	—	—
total	905	12.00 ± 0.97	7.81 ± 1.97	0.65 ± 0.12	—	—	—	—

Whales in the smaller size classes of both species were under-represented in the dataset as 10.7 m (35 ft) was the minimum catch length set by the International Whaling Commission (IWC), the regulatory body for the commercial whaling industry [50].

### 3.1. Inter-species differences

**Figure 3.**
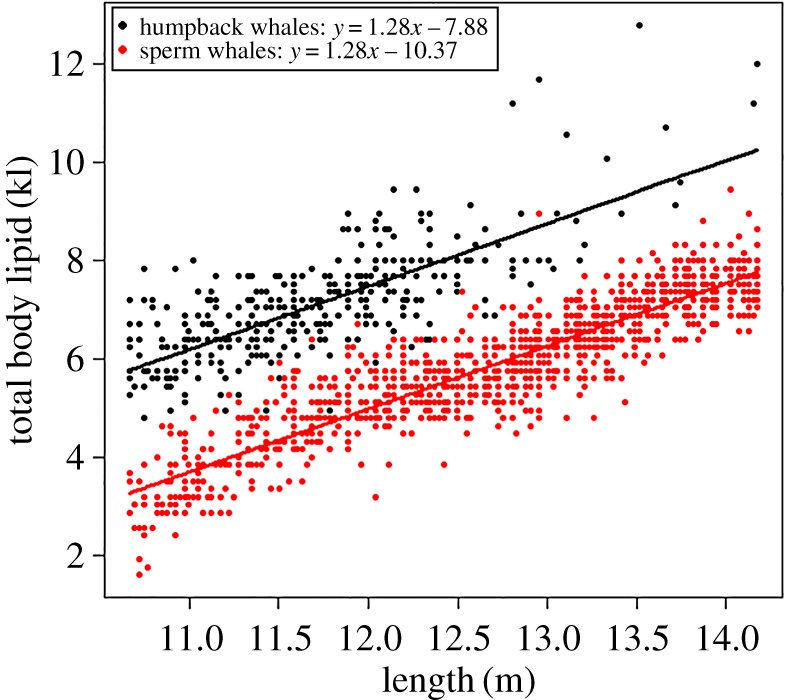
Relationship between total body lipid and the predictors in the top-ranked model (*length and species*) from the suite of models tested to explain total body lipid. Shown are the raw values (sperm whale data were truncated to the maximum length of humpback whales), fitted lines and regression equations of the top-ranked model for each species.

The top-ranked model included both species and length with no interaction term (*w*AICc = 0.54) and accounted for 81% of the variance explained (table 2). This demonstrated that total body lipid increased with length for both humpback and sperm whale males and the relationships had the same slope. Length (with the random factor year) accounted for 43% of the variation in body lipid; and the addition of species accounted for an additional 38% of the variation in body lipid (table 2). The relationship between body lipid and length for male humpback (*y* = 1.28*x *– 7.88) and sperm whales (*y* = 1.28*x *– 10.37) demonstrated that, for a given length, male humpback whales stored an average of 2.49 kl (15.6 barrels) (31.9–74.9%) more body lipid than male sperm whales (figure 3).

**Table 2.** Ranked (by AICc) linear mixed-effects models investigating the relationship between total body lipid and the explanatory fixed effects; species and length and the random effect of year. Shown are the number of parameters (*k*), the difference in AICc (Akaike's information criterion corrected for small samples) for each model from the top-ranked model (ΔAICc), the AICc model weight (*w*AICc), the difference in the BIC (Bayesian information criterion) (ΔBIC), the BIC model weight (*w*BIC) and the proportion of variance explained by fixed (*R*^2^_GLMM(m)_) and both fixed and random factors combined (*R*^2^_GLMM(c)_).

model	*k*	ΔAICc	*w*AICc	ΔBIC	*w*BIC	*R*^2^_GLMM(m)_	*R*^2^_GLMM(c)_
∼species + length + (1|year)	5	0	0.54	0	0.95	0.81	0.81
∼species × length + (1|year)	6	0.33	0.46	5.79	0.05	0.81	0.81
∼length + (1|year)	4	2011.75	<0.01	2006.29	<0.01	0.38	0.43
∼species + (1|year)	4	2697.89	<0.01	2692.43	<0.01	0.11	0.11
∼1 + (1|year)	3	2875.21	<0.01	2864.30	<0.01	<0.01	0.02

### 3.2. Intra-species differences

#### 3.2.1. Humpback whales

**Figure 4.**
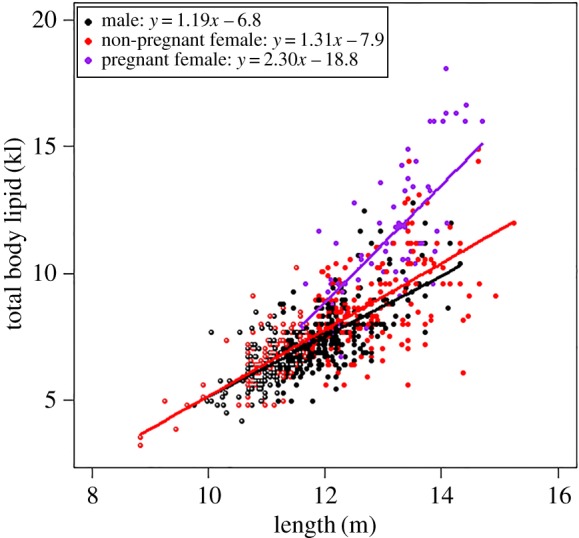
Relationship between humpback whale total body lipid and the predictors in the top-ranked model (*length and reproductive class*) from the suite of models tested to explain total body lipid. Shown are the raw values, fitted lines and regression equations for each reproductive class. Immature whales are colour-coded with non-filled centres: immature females with a red outline and immature males with a black outline.

The highest ranked model included length and reproductive class and the interaction between them (*w*AICc = 1) (table 3), demonstrating that there was a positive relationship between total body lipid and length in all reproductive classes. The slope differed according to reproductive class, with that of the pregnant females being the steepest (*y* = 2.30*x *– 18.8), followed by non-pregnant females (*y* = 1.31*x *– 7.9) and then males (*y* = 1.19*x *– 6.8) (figure 4).

**Table 3.** Ranked (by AICc) linear mixed-effects models investigating the relationship between humpback whale total body lipid and the fixed-effects length and reproductive class (class) and the random effect of year. See the caption for table 2 for a description of the table elements.

model	*k*	ΔAICc	*w*AICc	ΔBIC	*w*BIC	*R*^2^_GLMM(m)_	*R*^2^_GLMM(c)_
∼length × class + (1|year)	9	0	1.00	0	1.00	0.69	0.72
∼length + class + (1|year)	7	25.32	<0.01	15.78	<0.01	0.68	0.70
∼length + (1|year)	5	158.42	<0.01	139.32	<0.01	0.59	0.61
∼class + (1|year)	6	860.67	<0.01	846.35	<0.01	0.31	0.32
∼1 + (1|year)	4	1082.47	<0.01	1058.58	<0.01	<0.01	0.02

Pregnant female humpback whales stored up to twice as much body lipid as other whales of the same body length (figure 4), and on average stored 26.2% and 37.4% more body lipid (per metre) than non-pregnant females and mature males, respectively (table 1). Immature whales stored, on average, 7.9% and 14.7% less body lipid (per metre) than mature males and non-pregnant females, respectively (table 1).

**Figure 5.**
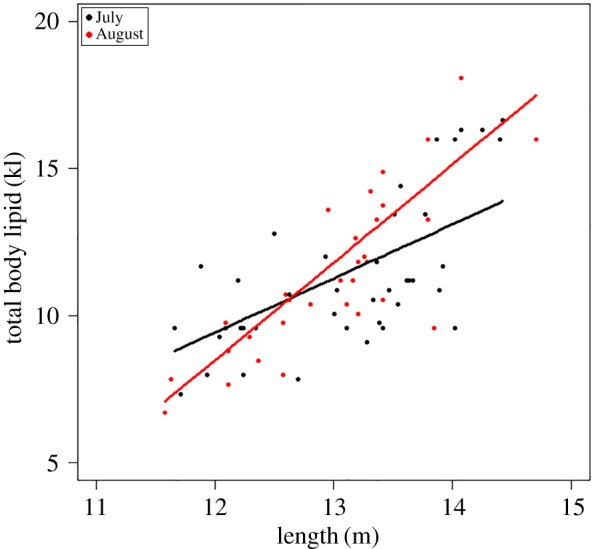
Relationship between humpback whale total body lipid and the predictors in the top-ranked model (*length and month*) from the suite of models tested to explain total body lipid for pregnant females. Shown are the raw values and fitted lines for the pregnant females in the months of July and August.

The relationship between body lipid and length of the pregnant females varied according to month (*w*AICc = 0.97) (table 4), with those sampled in August having a steeper slope than those sampled in July (figure 5). For the males, the model that included the interaction between length and month had equal support (within two AIC points) to the model with length alone when considering AICc; however, the model with length only had complete support when considering the BIC (table 4). Thus, there is little evidence for an effect of month. For the non-pregnant females and immature whales, the models that included length and month had majority support when considering AICc (AICc = 0.73 and 0.58, respectively). However, for the former, the BIC selected the model with length only (BIC = 0.85), and for the latter, the BIC values showed equal support for the model length + month (BIC = 0.45) and length only (BIC = 0.51). Thus there is minor evidence for an effect of month for the immature whales (slightly higher in month 7) (electronic supplementary material, figure S2 in appendix S2). The addition of month for all reproductive classes only accounted for an extra 2% of variance explained over and above length (table 4).

**Table 4.** Ranked (by AICc) linear mixed-effects models investigating the relationship, for each humpback whale reproductive class, between total body lipid and the fixed effects of length and month and the random effect of year. See the caption for table 2 for a description of the table elements.

model	*k*	ΔAICc	*w*AICc	ΔBIC	*w*BIC	*R*^2^_GLMM(m)_	*R*^2^_GLMM(c)_
males							
∼length × month + (1|year)	9	0	0.48	15.04	<0.01	0.47	0.57
∼length + (1|year)	5	0.13	0.46	0	1.00	0.45	0.55
∼length + month + (1|year)	7	4.23	0.06	11.71	<0.01	0.45	0.55
∼month + (1|year)	6	189.42	<0.01	193.10	<0.01	0.02	0.15
∼1 + (1|year)	4	197.50	<0.01	193.55	<0.01	<0.01	0.14
non-pregnant females							
∼length + month + (1|year)	7	0	0.73	3.45	0.15	0.34	0.39
∼length + (1|year)	5	3.07	0.16	0	0.85	0.32	0.36
∼length × month + (1|year)	9	3.72	0.11	13.61	0.01	0.34	0.39
∼1 + (1|year)	4	58.06	<0.01	51.70	<0.01	<0.01	0.01
∼month + (1|year)	6	60.63	<0.01	60.83	<0.01	<0.01	0.01
pregnant females							
∼length × month + (1|year)	7	0	0.97	0	0.84	0.55	0.74
∼length + (1|year)	5	7.26	0.03	3.59	0.14	0.53	0.73
∼length + month + (1|year)	6	9.65	0.01	7.85	0.02	0.52	0.71
∼1 + (1|year)	4	73.34	<0.01	67.73	<0.01	<0.01	<0.01
∼month + (1|year)	5	75.56	<0.01	71.89	<0.01	<0.01	<0.01
immature							
∼length + month + (1|year)	6	0	0.58	0.25	0.45	0.43	0.50
∼length × month + (1|year)	7	1.27	0.30	4.89	0.04	0.43	0.50
∼length + (1|year)	5	3.15	0.12	0	0.51	0.41	0.48
∼1 + (1|year)	4	125.38	<0.01	118.82	<0.01	<0.01	0.03
∼month + (1|year)	5	125.33	<0.01	122.38	<0.01	0.02	0.05

#### 3.2.2. Sperm whales

**Figure 6.**
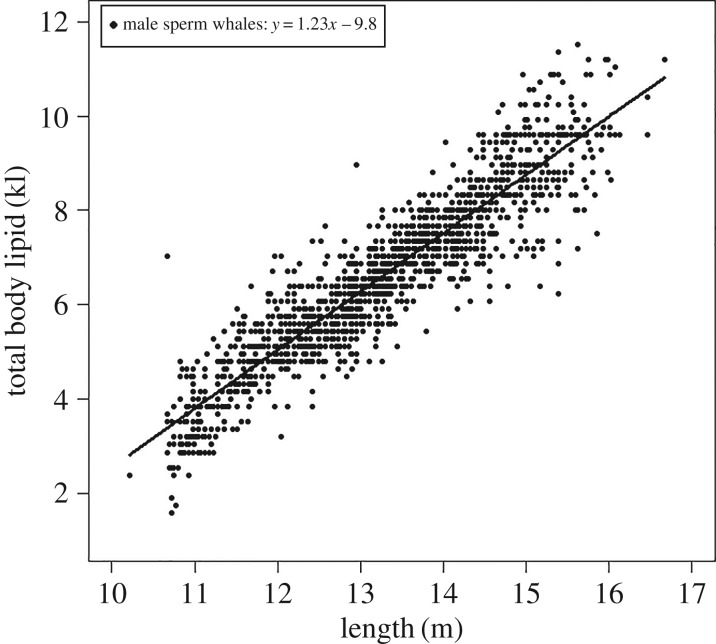
Relationship between sperm whale total body lipid (males only) and length (the top-ranked model from the suite of models tested to explain total body lipid). Shown are the raw values, fitted line and regression equation. Note: the equation provided here is slightly different from that provided for sperm whales in figure 3, as here the data were not truncated.

We did not find any evidence for a seasonal effect on oil yield for the male sperm whales with the model with length only having majority support (*w*BIC = 0.88) (table 5). The relationship between body lipid and length was described by the equation *y* = 1.23*x*−9.8 (figure 6). Note that the previous equation provided for sperm whales (figure 3) was that for truncated data (to match that of humpbacks in order to meet the assumptions of the model being fitted).

**Table 5.** Ranked (by AIC) linear mixed-effects models investigating the relationships between total body lipid of male sperm whales and the fixed effects of length and season and the random effect of year. See the caption for table 2 for a description of table elements.

model	*k*	ΔAICc	*w*AICc	ΔBIC	*w*BIC	*R*^2^_GLMM(m)_	*R*^2^_GLMM(c)_
∼length × season + (1|year)	10	0	1.00	4.25	0.10	0.86	0.86
∼length + season + (1|year)	7	20.74	<0.01	8.30	0.01	0.85	0.85
∼length + (1|year)	4	29.14	<0.01	0	0.88	0.85	0.85
∼season + (1|year)	6	3754.91	<0.01	3736.91	<0.01	0.02	0.02
∼1 + (1|year)	3	3785.29	<0.01	3750.57	<0.01	<0.01	<0.01

## 4. Discussion

Capital breeders are hypothesized to have higher energy stores than income breeders. Here, we found that capital breeding humpback whales stored 2.49 kl (31.9–74.9%) more body lipid than income breeding sperm whales of equivalent length. This not only demonstrates the substantial energy storage requirements of capital breeders, but also provides the first quantification of the energy required for male humpback whales to migrate to the coast of Australia from Antarctica. We also quantified, for the first time, the large lipid storage requirements of breeding female humpback whales and demonstrated that those arriving along the Australian coast later in the season had larger energy stores than those that arrived earlier. We suggest that this is the result of delaying migration to maximize energy stores, by increasing time in the Antarctic foraging grounds [32,37]. Importantly, we developed equations describing the relationship between body lipid and length that can be used both for bioenergetics modelling (e.g. [51–53]) and for predicting body lipid in the extensive historical whaling datasets held by the IWC [54], which typically include data on length and sex but not body lipid.

### 4.1. Inter-species comparisons

The difference of 2.49 kl of body lipid that we found between male humpback and sperm whales was constant, regardless of body length, indicating that the total energy required during the breeding fast is the same for individuals of all sizes. Consistent with the theory of mass-specific metabolism [15], this demonstrates that the relative energy required for migration is greater for smaller individuals than larger individuals.

As the energy stores of the BSD humpback whales decline through the breeding season [32], and those of sperm whales are stable throughout the year (table 5), the body lipid difference we found between the two species (2.49 kl) is probably an underestimate of the difference that would be expected when the humpbacks depart from their feeding grounds, carrying maximum energy stores. More specifically, the sampling location (Cheynes Beach Whaling Station) is located approximately 3000 km north of the BSD feeding grounds [32] and, contrary to that observed in some other humpback whale populations [55], there is no evidence of feeding during migration. Thus, the energy difference at the beginning of the migration would be expected to decrease continually throughout the season [32], until the humpback whales return to their feeding grounds and resume foraging. We suggest that by the time the humpback whales return to their Antarctic feeding grounds, the body lipid difference between humpback and sperm whales may be much smaller than 2.49 kl and possibly even close to zero. Unfortunately, it was not possible to investigate the energy stores of humpback whales on the return journey to their feeding grounds, as they do not pass Cheynes Beach Whaling Station on their southward migration, and oil yields of individual whales were not recorded at the other whaling stations along the Western Australian coast [32].

We can, however, estimate the maximum energy stores of humpback whales by using information from the Antarctic feeding grounds. Ash [36] reported that humpback whales accumulate 200 l (1.25 barrels) of body lipid each week in the feeding grounds, and by mid-February (the last sampling date) humpback whales of average length 12.8 m (42 ft) stored an average of 6.77 kl (42.3 barrels) of body lipid. Although departure dates from the feeding grounds are not well known, humpback whales are believed to begin departing in mid–late April [37] through to May [54]. Accumulating body lipid at a rate of 200 l per week (1.25 barrels per week) [36], a 12.8 m (42 ft) humpback would store between 8.57 and 8.97 kl (53.5–56 barrels) of lipid by mid–late April. This compares with 8.43 kl (52.7 barrels) for a male humpback whale of the same size, sampled at Cheynes Beach between June and August (using the equation we developed for male humpback whales; *y* = 1.19*x*−6.8). This indicates that the maximum difference in lipid storage between humpback and sperm whales could be 0.14–0.54 kl higher (per individual) than the 2.49 kl measured at Cheynes Beach Whaling Station, with the total difference between sperm and humpback whales being potentially as high as 3.03 kl. Although an interesting comparison, these figures must be interpreted with caution due to potential differences in factory efficiencies [32]. In addition, no information was provided by Ash about the sex of the whales processed in the Antarctic, or the variation in oil yield according to body length. Regardless of this, our study indicates that the energy required for the annual humpback whale migration is likely to be derived from at least 2.49 kl of body lipid.

In addition to sampling location, we must also account for differences in morphology between sperm and humpback whales, such as the oil-filled spermaceti organ of the sperm whale, which is not believed to play a role in energy storage [56–58]. Given that the sperm whale oil yield records from Cheynes Beach Whaling Station included spermaceti oil as well as sperm oil from the body [41], and that an average of 11% of the total oil produced from a sperm whale is spermaceti oil [57], the difference of 2.49 kl (15.6 barrels) of body lipid that we found between humpback and sperm whales is probably an underestimate of the difference in body lipid that is stored for energy utilization.

Interpreting differences in lipid stores between mysticetes and odontocetes, in terms of energetics, is a complex task due to differences in life history, behaviour, morphology and lipid storage. For example, humpback whales are a baleen whale specialized for long-distance migration. They have thick blubber that plays a role in thermoregulation, but is primarily an energy storage depot [21,28]. Sperm whales, by contrast, are specialized for deep diving. They have large heads that can weigh over one-third of their total body weight [57] and thick blubber that plays a role in structural support, thermoregulation and energy storage [28]. Previous comparisons between baleen whales and sperm whales have been based on body and tissue weights [28,59], and have demonstrated differences between slow-swimming baleen whales (humpback and right whales), fast-swimming baleen whales (blue, fin, sei and brydes) and sperm whales; more specifically, that: (i) the slow-swimming baleen whales are heavier per unit body length than sperm whales, while the fast-swimming baleen whales are lighter [59]; (ii) right whales contain greater proportions of blubber than sperm whales, while the fast-swimming baleen whales contain less [28,59] (unfortunately, no information was available for humpback whale blubber); (iii) the fast-swimming baleen whales have a higher proportion of muscle than sperm whales [28,59] and store the majority of their lipid stores in their muscle [16]. Sperm whales, in comparison, store almost no lipid in their muscle [28].

Measurement of total body lipid provides a useful method of comparing energy stores between species, as it enables inclusion of energy stores from all lipid depots, regardless of their function or storage capacity. Although this information is very valuable, there are some limitations of historical whaling data that need to be recognized: (i) the oil extracted from each whale is dependent on factory efficiency [32]; (ii) size classes and reproductive classes were not all equally represented as the whaling industry operated under regulations which included a minimum catch size of 10.7 m (35 ft) for humpback and sperm whales and also prohibited the taking of any calves and accompanying (lactating) females [50]; and (iii) at times, lengths of undersized whales were falsified to avoid infraction reports [32,40]. Despite these limitations, however, the catch records from Cheynes Beach Whaling Station provide an extremely valuable source of information on energy storage in cetaceans as they provide a measure of total body lipid for a large number of individual whales. Large, detailed datasets such as this are exceptionally rare and cannot be replicated in the modern era.

### 4.2. Intra-species comparisons

#### 4.2.1. Sperm whales

Sperm whales are income breeders that accrue regular energetic ‘income’ [39,60] and adjust their foraging rate according to energy demands [61]. The energy stores of the male sperm whales in this study were fairly consistent throughout the year, indicating that there was adequate food supply to satisfy their energetic demands year-round. There was no evidence of seasonal energy storage such as that found in other income breeders like long-finned pilot whales, which store energy in winter for use in reproduction during spring and summer [62].

Sperm whale weight, girth and blubber thickness have all been shown to increase with body length [19,28,59,63]. However, the relationship between blubber thickness and body length has at times been confounded by high individual variability and small sample size [30]. This study confirmed the high individual variability in energy stores found by Evans *et al.* [30], but the extensive nature of the dataset (1961 males) and the large range of body lengths (10.2 m (33.5 ft)–16.8 m (55 ft)) facilitated the identification of a positive relationship for the males. The lack of small whales in this study (no whales less than 10.2 m (33.5 ft)) places some uncertainty around the validity of the equation for the relationship between lipid and length for smaller individuals. However, the girth–length relationship of Lockyer [28] included smaller individuals (3.7 m (12 ft) in length) and suggests that this relationship is valid for all size classes.

#### 4.2.2. Humpback whales

Baleen whale energy stores have been shown to vary according to reproductive status [16,18,32,64–66], with pregnant blue and fin whales accumulating 20–25% more lipid than resting females [16], to satisfy the high energy demands of gestation and lactation [13,16]. Consistent with this, pregnant female humpback whales in this study stored an average of 26.2% and 37.4% more body lipid than non-pregnant females and males, respectively. Interestingly, our body lipid–length equations illustrate that the longer pregnant females stored relatively more energy than the shorter pregnant females, thus having more energy to transfer to their offspring [13]. Maximizing energy stores is critical for capital breeders, who must trade off their own body condition to maximize offspring survival [17,67]. Maternal energy stores have been shown to influence fecundity [68], fetal growth [67], weaning mass [69,70] and, thus, ultimately survival [70,71]. Large maternal energy stores enable longer fasting periods for the mothers [72,73], which in turn provide calves with a thermoregulatory benefit of longer duration in the warm waters of their breeding areas [15]. The relatively low energy stores of the smaller pregnant females in this study suggest that they will be more vulnerable to nutritional stress during the migration fast, particularly after commencing lactation [74], and will probably produce smaller calves with lower survival rates.

Hypothesis (i): mature, non-pregnant females will have variable energy stores due to their varying reproductive states.

As expected, the relative energy stores of the mature non-pregnant females exhibited high variation, due to their different reproductive states [16–18,23,66]. Females in this study presumably include individuals preparing for pregnancy, resting females and those that had recently terminated lactation. Lactating females generally have the lowest energy stores in the population [16–18,66], while those preparing for pregnancy tend to have the highest [23]. Although whaling industry regulations restricted the capture of lactating females accompanying calves [50] (thus preventing inclusion in this dataset), females with weaned calves were allowed to be captured. Given that lactation is generally terminated at the end of June [75], females that recently terminated lactation may have been caught during July and August and thus included in this study.

Hypothesis (ii): mature males will have higher energy stores than mature non-pregnant females, due to their need to compete for, and access, breeding females.

The relative energy stores of the males were similar to those of the non-pregnant females, suggesting that their energy demands over the breeding season are also similar. Contrary to our expectations, there was no evidence to suggest that the males stored higher energy reserves than females to fuel the competitive behaviours observed in the breeding grounds [34,76]. Male energy stores may be driven by a trade-off between energy accumulation in the feeding grounds and time maximization, and hence mating opportunities, in the breeding grounds [77]. It has been shown in other migratory species that males in good condition that reach the breeding grounds first have higher rates of breeding success [78,79]. Thus, the decision for male humpback whales to depart the feeding grounds may be based on accumulating a ‘sufficient’ or ‘ideal’ level of energy stores, rather than maximizing energy stores.

Hypothesis (iii): immature whales will have higher energy stores than mature males and mature non-pregnant females due to the high energy demands of growth.

It is generally accepted that immature mammals, including baleen whales, have greater energy demands than adults due to the energetic costs of body growth [35,80,81] and high mass-specific metabolic demands [12]. The relatively low energy stores of the immature whales in this study, in comparison with all other reproductive classes, suggest that they will be more prone to nutritional stress during the migration fast than mature whales. This appears to be supported by stranding data from the BSD population, which show that the majority of strandings along the migratory corridor are immature whales in generally poor body condition [82].

Hypothesis (iv): individuals sampled at Cheynes Beach Whaling Station later in the season will have higher energy stores than those sampled earlier, as they spend the summer accumulating and storing energy at a rate of 200 l per week, and all migrate at the same speed.

To gain a complete understanding of the effect of sampling date on energy stores, we must take into account reproductive status, as it is thought to influence residency times in the Antarctic foraging grounds [32,37,83], and thus drive the temporally staggered migration observed in humpback whale populations around the globe [32,77,83]. Our current understanding is that lactating females reside in the foraging grounds for approximately 4.5 months; immature animals, mature non-pregnant females and mature males stay for approximately 5.5 months; and pregnant females remain for about 6.5 months [37].

Our investigations of temporal differences in body lipid within each reproductive group provide evidence of individual variation in residency times in the feeding grounds coupled with an effect on energy stores. In particular, pregnant females sampled at Cheynes Beach Whaling Station later in the season had higher energy stores, on average, than those sampled earlier. As there is no evidence of differences in migration speed [83], arrival date along the Australian coast appears to reflect the departure date from the Antarctic, which in turn reflects residency times in the feeding grounds. Given that humpback whales accrue 200 l of body lipid per week in the feeding grounds [36], and it takes approximately three months for the entire migratory stream to pass Cheynes Beach Whaling Station *en route* to the breeding grounds [32], an increase in energy stores with sampling date is perhaps not surprising. Such an increase, however, has not been documented previously, and is the opposite of what may typically be expected in a capital breeding population. For example, Chittleborough [32] demonstrated that lipid stores of the BSD humpback whale population decreased during the breeding season, by sampling the population at a fixed point (Carnarvon Whaling Station: 24°53′ S, 113°38′ E; approximately 1100 km north of Cheynes Beach Whaling Station) during two different stages of the migration (northbound and southbound). This sampling regime described variation in energy stores *between* the northbound and southbound migratory streams that resulted from an estimated six-week difference in fasting duration [31,83], plus variation *within* the migratory streams due to variable residency times in the Antarctic feeding grounds. By contrast, our sampling regime describes variation in energy stores *within* the northbound migratory stream that are due solely to varying residency times in the Antarctic foraging grounds. Understanding the differences between these two sampling regimes is crucial, as they have significant effects on the results and thus on our interpretation of energy store variation during the breeding season.

Interestingly, our finding of an energetic benefit (for the pregnant females) from extra time in the feeding grounds appears to be restricted to the larger (longer) individuals. The relatively low body lipid stores of the smaller (shorter) pregnant females sampled later in the season may demonstrate their inferior energy storage capabilities due to small body size, size-related foraging efficiency [84] or perhaps late arrival in the feeding grounds. Given that small body size appears to increase vulnerability to nutritional stress during the migration fast [82], the additional energetic demands of reproduction [16] most likely renders the smaller breeding females (and their calves) as the most vulnerable component of the population during the annual migration.

## 5. Conclusion

The unique data summarized here demonstrate that capital breeding humpback whales store substantially more body lipid than income breeding sperm whales to fuel their annual migration, and that the energy stores of capital breeders are driven by a combination of body size, reproductive status and time spent in the feeding grounds. Pregnant female humpback whales delay their departure from the feeding grounds to maximize energy stores and satisfy the high costs of gestation and lactation. The smaller pregnant females, however, do not accumulate as much energy as the larger females, and are thus more vulnerable to nutritional stress during migration. Our study has provided new insights into the life-history strategies of large cetaceans, and the relationships we have quantified will be useful in developing ecosystem and bioenergetics models, and in understanding the potential impacts of environmental change. Moreover, the data we present here are particularly important given that such a large and detailed dataset on cetacean energy stores will probably never be collected again.

## Supplementary Material

Appendix S1. Summary Data (1952-1963). Here we provide summary data on body lengths of whales processed at Cheynes Beach Whaling Station between 1952 and 1963

## Supplementary Material

Appendix S2. Supplementary figures. Here we provide two additional figures from the analysis.

## Data accessibility

Summary data are available in appendix S1 in the electronic supplementary material. Raw data have been made available at the Australian Institute of Marine Science Data Centre (http://data.aims.gov.au/metadataviewer/uuid/3881b45c-2fdf-40c8-b0ce-dd6de3cb33b7) [85].
