# Supplementary material for: Quantifying the energy stores of capital breeding humpback whales and income breeding sperm whales using historical whaling records
Source: R Soc Open Sci. 2017 Mar 15;4(3):160290. doi: 10.1098/rsos.160290 (PMC5383807; doi:10.1098/rsos.160290)
Supplement: Appendix S1. Summary Data (1952-1963). Here we provide summary data on body lengths of whales processed at Cheynes Beach Whaling Station between 1952 and 1963 [file rsos160290supp1.docx]

*Royal Society Open Science*

**Supplementary material from “Quantifying the energy stores of capital breeding humpback whales and income breeding sperm whales using historical whaling records”**

Lyn G. Irvine, Michele Thums, Christine E. Hanson, Clive R. McMahon and Mark A. Hindell

Email: [Lynette.Irvine@utas.edu.au](mailto:Lynette.Irvine@utas.edu.au)

Appendix S1: Summary Data (1952-1963).

**Description**

Table S1. Here we provide summary data on body lengths of whales processed at Cheynes Beach Whaling Station between 1952 and 1963.

**Table S1.** Number and total body length (mean and sd) of humpback and sperm whales processed at Cheynes Beach Whaling Station, shown each year and each month between 1952 and 1963. F = females.

|  | Humpback whales (m) | | | | | | | | | | | | | Sperm whales (m) | | | | | | | | | | | |
| --- | --- | --- | --- | --- | --- | --- | --- | --- | --- | --- | --- | --- | --- | --- | --- | --- | --- | --- | --- | --- | --- | --- | --- | --- | --- |
|  | Reproductive group | | | | | | | | | | | | | Reproductive group | | | | | | | | | | | |
|  |  | All whales | | | Males | | | Pregnant F | | | Non pregnant F | | | All whales | | | Males | | | Pregnant F | | | Non pregnant F | | |
|  |  | n | mean | sd | n | mean | sd | n | mean | sd | n | mean | sd | n | mean | sd | n | mean | sd | n | mean | sd | n | mean | sd |
| Year | 1952 | 48 | 12.79 | 0.75 | 24 | 12.38 | 0.71 | 8 | 13.14 | 0.40 | 16 | 13.24 | 0.60 | 0 |  |  | 0 |  |  | 0 |  |  | 0 |  |  |
|  | 1953 | 75 | 12.23 | 1.10 | 41 | 12.07 | 0.94 | 12 | 13.23 | 0.46 | 22 | 11.96 | 1.35 | 0 |  |  | 0 |  |  | 0 |  |  | 0 |  |  |
|  | 1954 | 3 | 12.04 | 1.80 | 2 | 11.05 | 0.75 | 0 |  |  | 1 | 14.02 |  | 0 |  |  | 0 |  |  | 0 |  |  | 0 |  |  |
|  | 1955 | 122 | 12.24 | 1.06 | 78 | 12.14 | 0.79 | 3 | 12.60 | 0.93 | 41 | 12.42 | 1.45 | 5 | 14.05 | 2.52 | 4 | 14.88 | 1.97 | 0 |  |  | 1 | 10.74 |  |
|  | 1956 | 119 | 12.27 | 1.03 | 58 | 11.73 | 0.73 | 14 | 13.18 | 0.81 | 47 | 12.66 | 1.04 | 61 | 13.65 | 1.22 | 61 | 13.65 | 1.22 | 0 |  |  | 0 |  |  |
|  | 1957 | 101 | 12.05 | 0.83 | 58 | 11.81 | 0.65 | 13 | 13.00 | 0.93 | 30 | 12.11 | 0.84 | 139 | 13.06 | 1.56 | 122 | 13.36 | 1.41 | 4 | 10.75 | 0.03 | 13 | 10.89 | 0.26 |
|  | 1958 | 82 | 11.81 | 0.89 | 36 | 11.45 | 0.68 | 4 | 12.95 | 0.96 | 42 | 12.00 | 0.91 | 258 | 12.97 | 1.37 | 228 | 13.25 | 1.19 | 3 | 10.37 | 0.54 | 27 | 10.89 | 0.14 |
|  | 1959 | 159 | 12.08 | 1.02 | 75 | 11.80 | 0.85 | 21 | 13.11 | 0.72 | 63 | 12.07 | 1.08 | 137 | 13.41 | 1.30 | 137 | 13.41 | 1.30 | 0 |  |  | 0 |  |  |
|  | 1960 | 105 | 11.74 | 0.84 | 62 | 11.50 | 0.66 | 3 | 12.82 | 1.13 | 40 | 12.02 | 0.93 | 282 | 13.08 | 1.33 | 273 | 13.15 | 1.29 | 1 | 10.67 |  | 8 | 10.88 | 0.20 |
|  | 1961 | 105 | 11.57 | 0.78 | 53 | 11.39 | 0.61 | 0 |  |  | 52 | 11.74 | 0.89 | 454 | 13.25 | 1.15 | 450 | 13.27 | 1.13 | 2 | 11.15 | 0.61 | 2 | 10.49 | 0.39 |
|  | 1962 | 40 | 11.77 | 0.72 | 25 | 11.66 | 0.61 | 3 | 12.83 | 0.90 | 12 | 11.74 | 0.74 | 568 | 13.21 | 1.21 | 556 | 13.26 | 1.17 | 0 |  |  | 12 | 10.83 | 0.46 |
|  | 1963 | 2 | 11.93 | 0.48 | 0 |  |  | 0 |  |  | 2 | 11.93 | 0.48 | 135 | 13.15 | 1.23 | 134 | 13.16 | 1.23 | 1 | 11.20 |  | 0 |  |  |
|  | Total | 961 |  |  | 512 |  |  | 81 |  |  | 368 |  |  | 2039 |  |  | 1965 |  |  | 11 |  |  | 63 |  |  |
| Month | 1 | 0 |  |  | 0 |  |  | 0 |  |  | 0 |  |  | 29 | 12.62 | 1.41 | 24 | 13.02 | 1.20 | 2 | 10.68 | 0.02 | 3 | 10.74 | 0.03 |
|  | 2 | 0 |  |  | 0 |  |  | 0 |  |  | 0 |  |  | 1 | 12.09 | 0.00 | 1 | 12.09 |  | 0 |  |  | 0 |  |  |
|  | 3 | 0 |  |  | 0 |  |  | 0 |  |  | 0 |  |  | 111 | 13.08 | 1.19 | 108 | 13.14 | 1.15 | 0 |  |  | 3 | 11.09 | 0.51 |
|  | 4 | 0 |  |  | 0 |  |  | 0 |  |  | 0 |  |  | 245 | 12.98 | 1.30 | 237 | 13.05 | 1.26 | 0 |  |  | 8 | 10.83 | 0.16 |
|  | 5 | 3 | 11.36 | 1.37 | 1 | 10.80 |  | 0 |  |  | 2 | 11.65 | 1.81 | 205 | 12.94 | 1.29 | 186 | 13.14 | 1.19 | 3 | 11.15 | 0.46 | 16 | 10.99 | 0.24 |
|  | 6 | 257 | 11.85 | 1.08 | 120 | 11.53 | 0.72 | 2 | 13.18 | 1.29 | 135 | 12.12 | 1.25 | 95 | 12.97 | 1.28 | 90 | 13.10 | 1.19 | 0 |  |  | 5 | 10.65 | 0.34 |
|  | 7 | 574 | 12.01 | 0.92 | 330 | 11.78 | 0.78 | 44 | 13.12 | 0.77 | 200 | 12.15 | 0.97 | 61 | 13.09 | 1.12 | 60 | 13.14 | 1.07 | 0 |  |  | 1 | 10.29 |  |
|  | 8 | 127 | 12.54 | 0.83 | 61 | 12.29 | 0.69 | 35 | 13.02 | 0.68 | 31 | 12.48 | 1.01 | 222 | 13.13 | 1.17 | 217 | 13.19 | 1.13 | 0 |  |  | 5 | 10.85 | 0.05 |
|  | 9 | 0 |  |  | 0 | 0 |  | 0 |  |  | 0 |  |  | 335 | 13.42 | 1.27 | 330 | 13.46 | 1.24 | 1 | 10.72 |  | 4 | 10.73 | 0.35 |
|  | 10 | 0 |  |  | 0 | 0 |  | 0 |  |  | 0 |  |  | 346 | 13.26 | 1.27 | 342 | 13.29 | 1.25 | 1 | 10.80 |  | 3 | 10.75 | 0.05 |
|  | 11 | 0 |  |  | 0 | 0 |  | 0 |  |  | 0 |  |  | 301 | 13.36 | 1.34 | 284 | 13.52 | 1.22 | 4 | 10.49 | 0.49 | 13 | 10.92 | 0.24 |
|  | 12 | 0 |  |  | 0 | 0 |  | 0 |  |  | 0 |  |  | 88 | 13.20 | 1.28 | 86 | 13.25 | 1.23 | 0 |  |  | 2 | 10.72 | 0.04 |
|  | Total | 961 |  |  | 512 |  |  | 81 |  |  | 368 |  |  | 2039 |  |  | 1965 |  |  | 11 |  |  | 63 |  |  |
