# Supplementary material for: Quantifying the energy stores of capital breeding humpback whales and income breeding sperm whales using historical whaling records
Source: R Soc Open Sci. 2017 Mar 15;4(3):160290. doi: 10.1098/rsos.160290 (PMC5383807; doi:10.1098/rsos.160290)
Supplement: Appendix S2. Supplementary figures. Here we provide two additional figures from the analysis. [file rsos160290supp2.docx]

*Royal Society Open Science*

**Supplementary material from “Quantifying the energy stores of capital breeding humpback whales and income breeding sperm whales using historical whaling records”**

Lyn G. Irvine, Michele Thums, Christine E. Hanson, Clive R. McMahon and Mark A. Hindell

Email: [Lynette.Irvine@utas.edu.au](mailto:Lynette.Irvine@utas.edu.au)

**Appendix S2: Supplementary analyses**

**Description**

This appendix contains the following supplementary figures:

**Figure S1:** Frequency distribution of total body length of humpback and sperm whales

**Figure S2:** Effect of length and month on humpback whale lipid stores


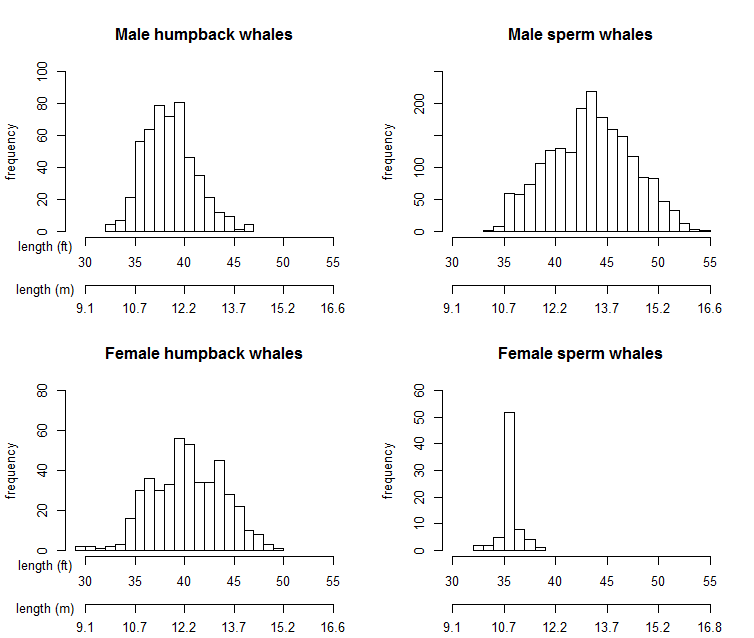


**Figure S1.** Frequency distribution of total body length of humpback and sperm whales sampled at Cheynes Beach Whaling Station between 1952 and 1963. The two x axes on each plot illustrate length in two different scales: top axis (ft) - as originally measured; bottom axis (m) - after conversion into SI units.


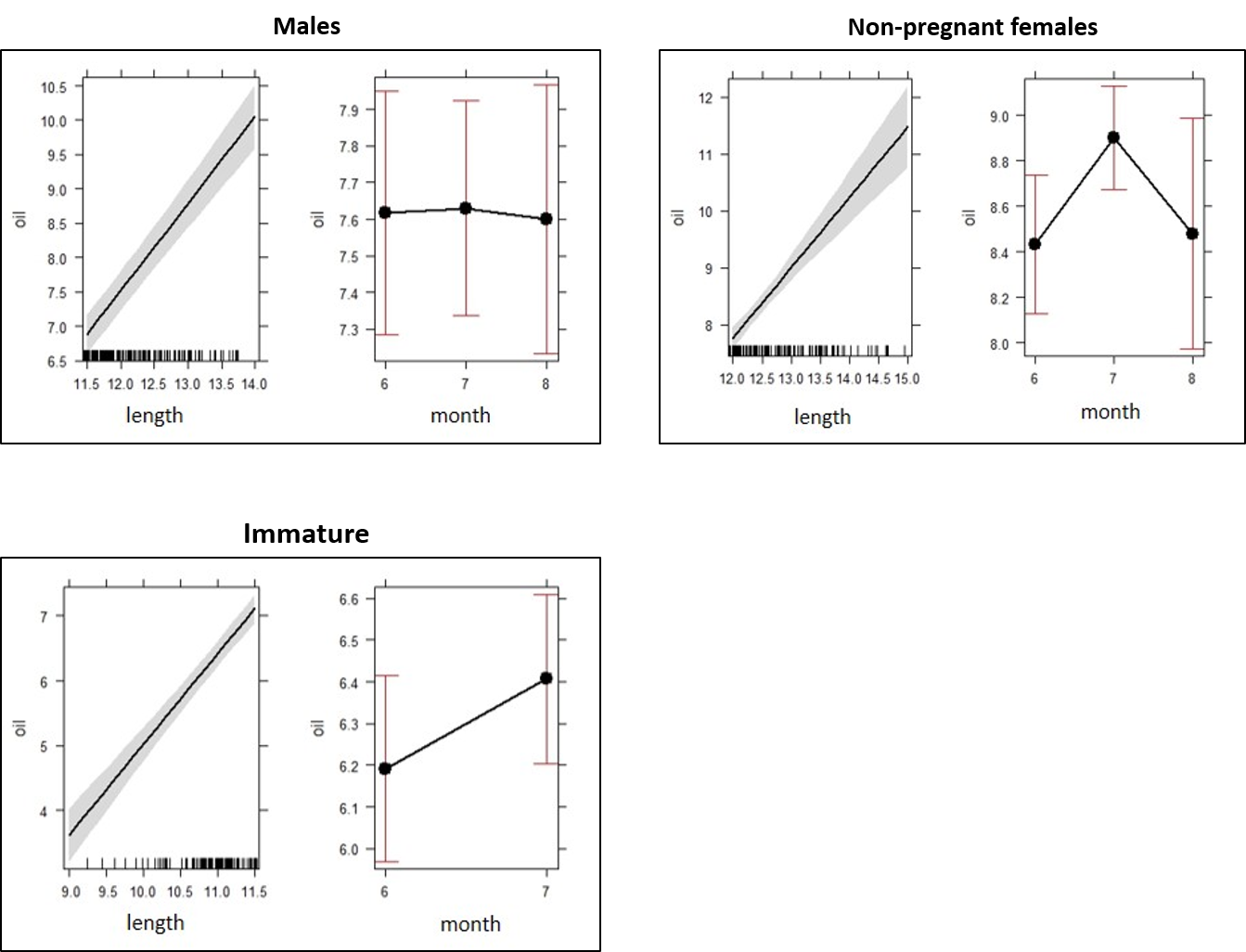


**Figure S2.** Relationship between humpback whale total body lipid and the predictors (*length and month*) for each reproductive class.
